# The Functions of Government*From a paper read a meeting of the State Society of Social Hygiene at Austin, Texas, January, 1910.

**Published:** 1912-07

**Authors:** R. L. Batts

**Affiliations:** Austin, Texas


### Department of Eugenics.

### THE TEXAS STATE SOCIETY OF SOCIAL HYGIENE.

Headquarters: San Antonio, Texas.

Dr. Malone Duggan, President, San Antonio.
Dr. F. E. Daniel, 1st Vice President, Austin.
Prof. A. Caswell Ellis, 2d Vice President, Austin.
Mrs. Eli Hertzberg, 3d Vice President, San Antonio.
Dr. Theo. Y. Hull, Secretary, San Antonio.
Mr. W. L. Evans, Treasurer, San Antonio.

EXECUTIVE COMMITTEE.

| | | |
|---|---|---|
| Dr. Malone Duggan, Chm. | Hon. J. C. Willacy. | Dr. W. A. King. |
| Dr. Theo. Y. Hull, Sec'y. | Mr. W. L. Evans. | Dr. J. W. Torbett. |
| Dr. W. F. McCaleb. | Dr. Frank D. Boyd. | Dr. F. C. Walsh. |
| Hon. C. H. Jenkins. | Dr. J. R. Nichols. | Dr. Joe Reuss. |
| Rev. A. W. S. Garden. | Dr. J. M. McCutchan. | Dr. Frank Paschal. |

### The Functions of Government.*

BY R. L. BATTS, AUSTIN, TEXAS.

Government is essential; essential not alone to welfare but to existence. Wherever men come together, in whatever number, government naturally arises. It is in operation before its existence is recognized, and its existence is known before its character is formulated into words.

There can be no definite limits for governmental action. Progress is a law of life. The goal is not so discouragingly near that we may see it or know it. But going to an end is itself an end. Man must move or die. And the government he makes must progress or decay. Progress does not necessarily consist in extending its limits, but may be an increase of efficiency within the limits.

The happiness and welfare of men is an all-sufficient reason for the existence of government. There is no other reason and

*From a paper read a meeting of the State Society of Social Hygiene at Austin, Texas, January, 1910.

no other excuse. The failures to accomplish these fundamental ends have been so frequent and so long continued that this plain truth is usually overlooked. It illustrates the difficulty of seeing the obvious. So entirely is the government's reason for being forgotten that its essential character is misunderstood. There is a feeling that it is something intangible; that it is powerful, implacable, merciless; that it is a monster of mysterious power; awe-inspiring; fearsome. The feeling is less difficult to understand than to excuse. It exists even with reference to our own government, which has behind it no centuries of misdeeds to excite horror or obscure its origin. The time and manner of its birth is known to children. Our immediate ancestors made it for their common defense and general welfare, and for ours. Yet we look upon it as a thing apart from the people; as something with which we are to wage an unequal contest; as something with powers and functions not to be understood except by a greatly gifted, highly specialized, peculiarly privileged class.

The fault lies in the failure to recognize the fundamental fact that government is by men for men; that when it no longer serves them it has been subverted; that the only remedy for its deficiencies lies in the hands of those whose physical strength, whose intellectual capacity and whose moral force makes its existence possible, and that when it is no longer supported by the consent of the governed it disintegrates.

We are the State. We should not be ignorant of it. We should not fear it. We can make it what we will. If it is oppressive we are but permitting it to be used by some of our fellows to bring about conditions it is designed to prevent. We do not need a new government. We do not need a revolution. We need but a recognition of the fact that government is a device—essentially simple—by men to protect themselves and help themselves, and that its existence and administration is in no sense dependent upon a privileged and specialized class.

Excepting those to whom public office is a profession (and these are distinguished from those to whom public service is an occupation) and the limited number who are enabled under a plea of protection or patriotism to become parasites, the people do not desire of the government anything but the common good. They are satisfied if the government be not affirmatively bad. Herein they are derelict. It can not be bad except by their acquiescence. And why should it not be an affirmative force for good?

We are predacious, wherein we are as the animals; we are unselfish, wherein we are of the Divine essence. Our aim should be protection from the one instinct, encouragement of the other. For the accomplishment of these greatest ends there should be used our most efficacious means—our governmental organization.

The altruism of the Christ is the surest basis for individual well-being and happiness. No scheme of theology—no system of philosophy—can supply that which comes from unselfishness, from the cheerful performance of those simple duties that affect our fellows. With these well done, and with appreciation of the beauty of the things that are around us and under and far above, and of the infinite greatness of the Maker who fixed their marvelous qualities, neither life should be without its great happiness nor death without its pleasant anticipations.

Nor is there a better basis than this Christian altruism for government. The very practical man does not mistake mankind, and he does not expect to speedily have things as they should be. But he knows that every unselfish act upon his part for the public good will have its little effect if not its great effect, and that there is nothing so contagious as goodly deeds.

And, after all, the principal difficulty lies in the failure to recognize that there is no especial difficulty in correcting the faults and supplying the deficiencies of government.

Manhood is the government's concern. Or, rather, manhood is man's concern and the government one of his agencies therefor. Complete manhood may not be accomplished when men must live always in danger of loss of life and property. The State must give safety. Complete manhood may not be accomplished when that which a man earns is withheld. The State must give redress. The State may not long delay this justice. Much less may the State take the product of industry and give to those industrious only in importunity.

The first function of government is the maintenance of these and the other fundamental rights of men, but if it stops with this it stops far short of its great possibilities for good. Many enterprises for the public good are beyond individual endeavor. Besides, philanthropy is not without its evils. As no man should shirk his public duties, so each may feel that he has a right to that provided for all. It is best for men to do for themselves, and the government is their agency. The necessity for philanthropy largely disappears with the obstacles to opportunity. Nothing so well advertises the viciousness of the laws as the necessity for providing for the poor. There will always be occasion for the sweet voice and gentle hand of charity. But perfect laws and complete justice will leave little to private philanthropy.

The most notable example to this time of the exercise of governmental functions not involving prohibition and punishment is public education. It is based upon the idea that good government can not consist with ignorance. And this is true not alone because ignorance is inefficient but because government is that men may be properly and normally developed, and mental growth is a part of such development. The direct effect of this governmental policy is improvement in all governmental activities, and a general increase in the productive capacity and the capacity for happiness and progress.

The physical health is not less a common concern than the mental development. To this the education provided by the State contributes substantially, but government abrogates one of its most useful functions when it fails in further effective solicitude for the public health. While its best work has been in the aid given to higher education, manifested in the development of the science of surgery and the progressive approximation of medicine to a science, and while the State has done good work in keeping disease from its citizens, the people have yet in their government vast capacities for good in helping their fellows to battle with disease. They have been strangely negligent in providing for the safety of the many and giving hope to the unfortunate few by providing ample facilities for the cure of those afflicted with tuberculosis. Less important, but quite important, is the case of the lepers. When the Texas State Constitution was adopted thirty-five years ago, the necessity of establishing an asylum for those afflicted with alcoholism was recognized, but these unfortunates are yet to receive the helping hand of their fellows.

The efforts of governments to deal directly with so delicate a thing as the moral qualities of man have not been eminently successful. Morality is not a fixed quality. Christ, supplementing the Commandments, announced a system of ethics, the perfection of which is not questioned, but mankind has not progressed to the point where that system furnishes the rule of conduct.

Man is not machine made, nor made by legislation, nor made by governmental institutions. He is the inheritor of his forebears, but he is principally the maker of himself. The fine qualities of courage, honor, fidelity, kindness, cleanness, temperance, these come up out of his life, and no government may give them.

But neither this fundamental fact nor the decision, doubtless wise, to segregate the State from the church leaves the government without labors in the domain of morals. The prohibitions and punishments of the penal code have doubtless some effect upon the conduct of men, but have little to do with the morals of men.

Still there is opportunity for doing much in the field of morals in addition to that now being accomplished in aiding the mental and physical development.

The physical, mental and moral development of the race could be facilitated by a closer alliance with science, and the use of the means disclosed by scientific investigation to prevent progressive degeneracy and congenital criminality. In unnumbered other ways it is a machinery the people could use for their good. Its field is as broad as human thought and action.

The government is ours. It is for us. It is that we may be a happy, prosperous, progressive people; that we may live the normal life of normal men. Let us use it.

